# Do Xylylenes Isomerize in Pyrolysis?

**DOI:** 10.1002/cphc.202000317

**Published:** 2020-06-23

**Authors:** Florian Hirsch, Kai Pachner, Ingo Fischer, Kevin Issler, Jens Petersen, Roland Mitric, Sjors Bakels, Anouk M. Rijs

**Affiliations:** ^1^ Institute for Physical and Theoretical Chemistry University of Würzburg Am Hubland 97074 Würzburg Germany; ^2^ Radboud University Institute for Molecules and Materials FELIX Laboratory Toernooiveld 7 6525 ED Nijmegen, The Netherlands

**Keywords:** biradicals, high-temperature chemistry, IR spectroscopy, pyrolysis, xylylene

## Abstract

We report infrared spectra of xylylene isomers in the gas phase, using free electron laser (FEL) radiation. All xylylenes were generated by flash pyrolysis. The IR spectra were obtained by monitoring the ion dip signal, using a IR/UV double resonance scheme. A gas phase IR spectrum of para‐xylylene  was recorded, whereas ortho‐ and meta‐xylylene were found to partially rearrange to benzocyclobutene and styrene. Computations of the UV oscillator strength  for all molecules were carried out and provde an explanation for the observation of the isomerization products.

## Graphical abstract

**A gas phase IR spectrum** of para‐xylylene was recorded by IR/UV ion dip spectroscopy. The molecule was generated by flash pyrolysis. Ortho‐ and meta‐xylylene partially rearrange to benzocyclobutene and styrene. Their detection can be explained by the oscillator strength of the UV transition.

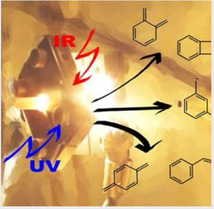

The interest in the chemistry of isolated xylylenes **1**–**3** originates from their biradicalic character,^1^ but also from their role in combustion. Here, they appear as high temperature decomposition products of xylenes, which are used as anti‐knock fuel additives.^2^ Spectroscopic work was mostly carried out using matrix‐isolation techniques,^3^ but in particular for comparison with computations, gas‐phase experiments are advantageous. Threshold‐photoelectron spectra of **1**–**3** produced by pyrolysis of suitable precursors were reported by Hemberger and co‐workers.^4^ The data gave structural and thermochemical information and yielded insight into the high‐temperature chemistry of xylylenes. Using femtosecond time‐resolved photoelectron spectroscopy, we recently investigated the excited‐state dynamics of para‐xylylene **1** generated by pyrolysis of [2,2]paracyclophane **4**. Detailed information on the excited state dynamics was extracted by the comparison with nonadiabatic trajectory simulations.^5^ The third harmonic of a Ti : Sa laser at 267 nm was employed for excitation. However, in such time‐resolved experiments it is important to start with an isomerically pure sample and to rule out isomerization in the pyrolysis reactor. It is the goal of the present work to test whether pyrolysis yields xylylenes pure enough for experiments relying on UV excitation around 265–267 nm, a typical pump wavelength in fs‐experiments.

IR‐UV ion dip spectroscopy is an excellent method to characterize isomerization, because it combines the mass‐selectivity of UV photoionization mass spectrometry with the structural sensitivity of IR spectroscopy.^6^ Here, molecules are ionized in a [1+1] process at a fixed UV wavelength. When an IR‐active mode of the molecule is excited by a laser that appears slightly earlier in time, the molecular ground state is depopulated and the ion signal decreases. Since an intense IR source is required to excite dilute samples in the mid‐IR fingerprint region, the experiments employed radiation from a free electron laser (FEL). The concept has been demonstrated in previous work on the isomers of the xylyl radical,^7^ but also on the isomer‐specific characterization of polycyclic aromatic hydrocarbons (PAH) formed in bimolecular reactions of radicals.^8^ We want to point out that a sufficient oscillator strength at the chosen UV wavelength is indispensable for an IR‐UV experiment, but this condition is often fulfilled for molecules with an aromatic chromophore. As shown in Scheme 1, in the present work the precursors [2,2]paracyclophane **4**, 3‐isochromanone **5** and 1,3‐bisbromomethylbenzene **6** have been applied to produce para‐ (**1**), ortho‐ (**2**) and meta‐(**3**) xylylene in a free jet. The relative energies of the relevant C_8_H_8_ isomers are given in brackets, with para‐xylylene arbitrarily set to 0. The values were taken from G3SX computations.4c, 9

**Scheme 1:**
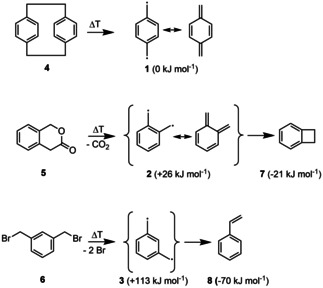
Pyrolysis of [2,2]paracyclophane **4**, 3‐isochromanone **5** and 1,3‐bisbromomethylbenzene **6** was employed to generate the three isomers of xylylene in a free jet. The energies relative to para‐xylylene were taken from the computations in Ref.4c, 9 Resonance structures are given for ortho‐ and para‐xylylene.

The [1+1] photoionization mass spectra at 265 nm showed full conversion of all three precursors and clean formation of the C_8_H_8_^+^ molecular ion at m/z=104, as well as some fragments that originate from dissociative photoionization. Note that experiments were conducted under conditions that minimized bimolecular reactions in the pyrolysis reactor, in contrast to earlier studies.^8^

IR/UV spectra of m/z=104, generated from **4**, are presented in Figure 1. The experimental IR spectrum (black line) is compared to a computed spectrum of para‐xylylene (blue line), obtained from DFT calculations at the B3LYP/6‐311++G**level of theory.^10^ For all xylylenes, the vibrational modes were calculated taking anharmonicity into account and their wavenumbers were not corrected by a scaling factor. The excellent agreement demonstrates that under the experimental conditions only **1** is observed. The experimental spectrum, which is dominated by a deformation mode at 864 cm^−1^ and the CC mode at 1583 cm^−1^ agrees well with a previous IR‐spectrum of **1** recorded in a rare gas matrix.3a, 3b Although all bands represent fundamentals, including anharmonicity in the computations leads to more accurate peak positions and a better description of the relative band intensities. The effect of anharmonicity on the mid‐IR spectra was recently systematically explored for polycyclic aromatic hydrocarbons.^11^

**Figure 1:**
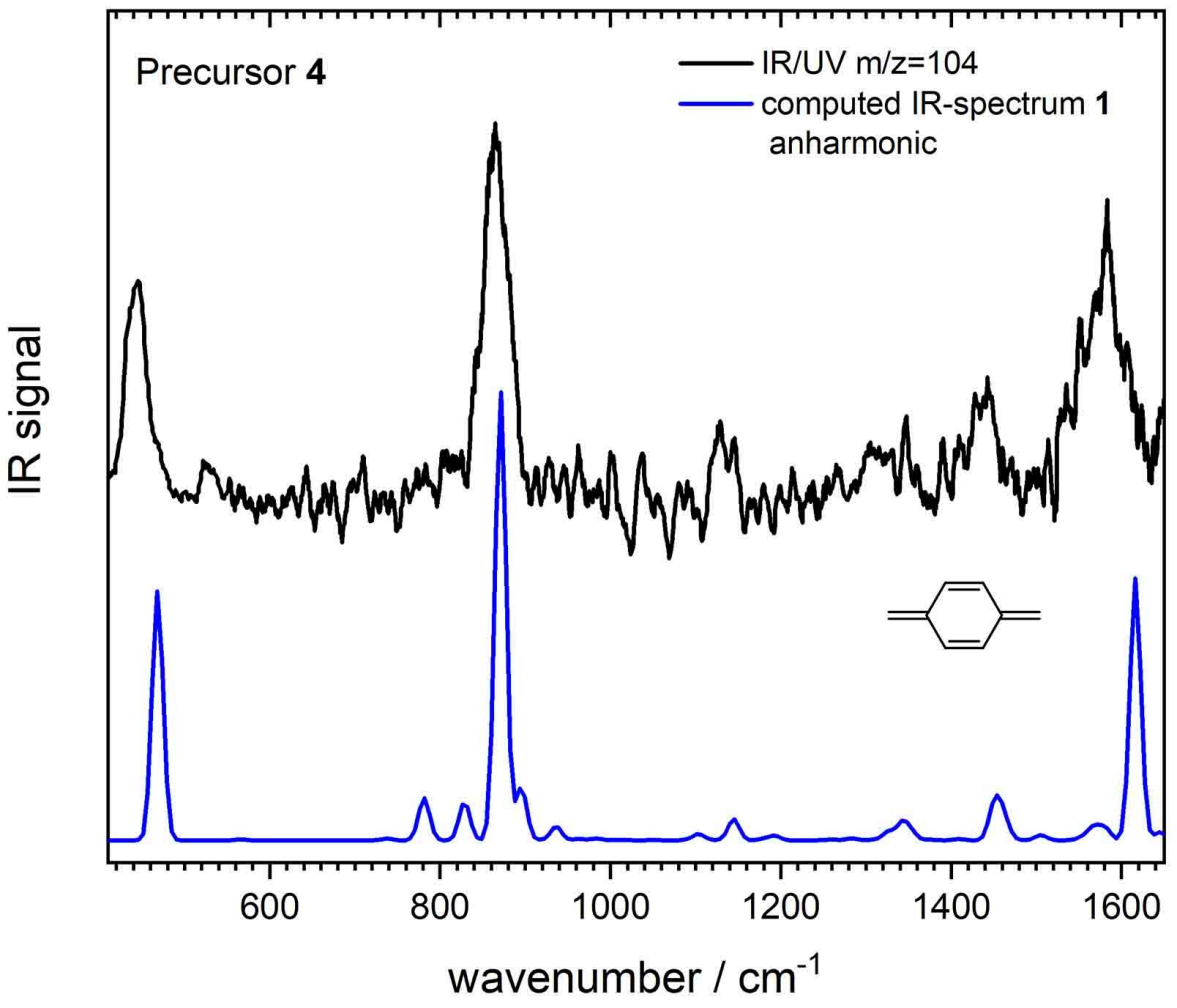
IR‐UV spectrum of mass 104 generated in the pyrolysis of **4** (upper trace). The simulated spectrum based on anharmonic calculations (lower trace) confirms that para‐xylylene is formed.

Figure 2 depicts the IR‐UV spectrum of m/z=104, generated from **5**. The simulated IR‐spectrum of **2** (blue spectrum, bottom trace) does not match the experimental spectrum in the top trace. Note that a C_2_ symmetric structure was found to be the energy minimum of the electronic ground state with the CH_2_ groups slightly twisted out of ring plane. This distortion reduces interactions between the hydrogen atoms. Evidently, none of the major bands is represented in the simulation. Instead the computed harmonic spectrum of the isomer benzocyclobutene **7** agrees well with the experimental one. Due to this excellent agreement, we did not carry out further computations including anharmonicity for **7**. Isomerization via [2+2] cycloaddition from **2** to **7** is exothermic by 0.48 eV and proceeds over a barrier of only 0.17 eV.^12^ The reaction is thus facile at elevated temperatures.

**Figure 2:**
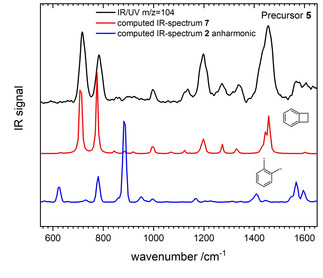
IR/UV spectrum of mass 104 generated in the pyrolysis of **5** (upper trace). A comparison with computed spectra shows that benzocyclobutene **7** is the carrier of the spectrum (middle trace), rather than ortho‐xylylene.

A similar situation is encountered for the pyrolysis of **6**, see Figure 3. The bands at 705 cm^−1^, 780 cm^−1^ and 910 cm^−1^ are not properly represented in the simulated spectra of meta‐xylylene **3** (blue line). Instead a harmonic simulation for styrene **8** (red line) matches the spectrum very well. Again, the agreement did not warrant further computations including anharmonicity for **8**.

**Figure 3:**
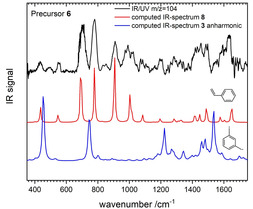
IR‐UV spectrum of mass 104 generated in the pyrolysis of **6** (top trace). A comparison with computed spectra shows that styrene **8** is the carrier of the spectrum (red). Isomer **3** does not fit, even when anharmonic corrections are included (blue).

Note that styrene is the lowest energy C_8_H_8_ isomer and for the isomerization from **3** to **8** a heat of reaction Δ_R_H(0 K)=−183 kJ ⋅ mol^−1^ is obtained from G3 computations, i. e. the reaction is strongly exothermic.4a Interestingly styrene has been observed in the combustion of xylenes,^13^ and it was suggested that this reaction proceeds via xylylene intermediates. Most likely the reaction from **3** to **8** proceeds via **2**. For the isomerization from **2** to **8** a barrier of 242 kJ ⋅ mol^−1^ has been computed.4a

Photoelectron spectra of all three xylylenes have been reported,4b, 4c so a generation by pyrolysis is possible and the observation of isomerization might be surprising at first glance. However, there are two other factors that have to be considered, (a) the choice of precursor and (b) the strength of the UV transitions. The previous photoionization study of **3** used a bisiodomethylbenzene precursor, which requires lower pyrolysis power for conversion and might result in less isomerization.4c Furthermore, in experiments using synchrotron radiation a continuous beam is used, whereas the FEL experiments described here require a pulsed jet. Due to its low vapour pressure we did not obtain sufficient signal intensity from the bis‐iodo compound for our experiments. But more importantly, in any experiment that involves excited electronic states, their oscillator strength *f* becomes relevant.

We therefore reviewed the available spectroscopic data and computed vertical excitation energies and oscillator strengths for several molecules relevant in the present work, as compiled in Table 1. The measured UV absorption spectrum of **1** features a broad band between 4.2 and 4.7 eV3a which is assigned to the bright ππ* transition to a ^1^B_2u_ state with a large oscillator strength close to 1 (cf. Ref.5a and Table 1). Notice that the spectral origin is expected to be red‐shifted with respect to the vertical excitation energy. Indeed, TDDFT calculations at the B3LYP/6‐311++G** level show that the transition between the lowest vibrational levels of the ground and the B_2u_ state lies 0.36 eV below the vertical excitation. Thus **1** is efficiently excited at 265 nm (4.68 eV). The situation changes in ortho‐xylylene **2**. A bright ππ* transition (*f*≈0.15*)* at lower energy (3.87 eV) was computed for **2**. Several dipole‐allowed transitions are present around 5 eV, corresponding to ππ* and Rydberg‐states, but their oscillator strength is almost two orders of magnitude smaller. In contrast, for benzocyclobutene (**7**) a bright vertical ππ* transition with *f ≈*0.017 is computed at 4.97 eV. Taking again the red‐shift of the spectral origin into account, for **7**, UV‐absorption around 265 nm is estimated to be an order of magnitude larger than for **2**. For meta‐xylylene **3**, measured absorption is appreciable in the range of 4.3–4.5 eV and strong between 5.2–5.6 eV.3c Theoretically, besides a variety of almost dark low‐lying ππ* and Rydberg transitions, a bright ππ* state (*f* ≈0.13) was computed at 4.97 eV and is assigned to the lower‐energy experimental band. In styrene (**8**), two electronic absorption bands have been experimentally found in the relevant energy range: a weak one between 4.3‐4.5 eV^16^ and a strong one starting at 4.87 eV and maximizing around 5.2 eV,^17^ which can both be assigned by theory to ππ* states (cf. the vertical transitions presented in Table 1 and the calculations given in Ref.^18^). The styrene band starting at 4.87 eV is considerably closer to the employed laser wavelength than the strong band in m‐xylylene. It is therefore likely that benzocyclobutene and styrene dominate the IR‐UV spectra due to their higher oscillator strengths and/or more resonant excitation energies.

**Table 1:** Computed vertical transitions to low‐lying singly excited electronic states, using EOM‐CCSD^14^ with the aug‐cc‐pVDZ basis set^15^ . For the assignment of C_2_/C_2v_ symmetry species, the C_2_ axis is chosen as z‐axis, and the x‐axis is perpendicular to the ring plane. For p‐xylylene, the molecule lies in the xy‐plane with the y‐axis containing the terminal methylene carbons. For styrene, the optimized structure has no symmetry as it slightly deviates from planarity. The given symmetry labels apply approximately for a planarized C_s_ geometry.

	State symmetry	ΔE_vert_[eV]	*f*	Character
	^1^A_1g_	3.72	0.000	ππ*^23^
p‐Xylylene (D_2h_)	^1^B_2u_	4.91	0.986	ππ*
	^1^B_1g_	5.15	0.000	ππ*
	^1^B_1u_	5.31	0.002	Ry‐3s
o‐Xylylene (C_2_)	^1^B	3.87	0.153	ππ*
	^1^A	5.13	0.001	Ry‐3s
	^1^B	5.31	0.003	ππ*
	^1^A	5.50	0.003	Ry‐3p_z_
Benzocyclobutene	^1^A_1_	4.97	0.017	ππ*
(C_2v_)	^1^B_1_	6.08	0.008	Ry‐3s
m‐Xylylene (C_2v_)	^3^A_1_	3.59	0.001	ππ*
	^3^B_2_	3.92	0.001	ππ*
	^3^A_1_	4.44	0.002	ππ*
	^3^B_1_	4.72	0.000	Ry‐3s
	^3^B_2_	4.79	0.001	ππ*
	^3^A_2_	4.86	0.009	Ry‐3p_y_
	^3^A_1_	4.97	0.127	ππ*
	^3^B_1_	5.21	0.000	Ry‐3p_z_
Styrene (C_1_)	(^1^A‘)	4.79	0.007	ππ*
	(^1^A‘)	5.50	0.370	ππ*
	(^1^A‘‘)	5.92	0.011	Ry‐3 s

In summary, the IR‐UV ion dip spectra show that para‐xylylene can be cleanly produced and excited at 265 nm, whereas ortho‐ and meta‐xylylene isomerize at least partially in the pyrolysis. Future time‐resolved experiments in the UV should therefore take this isomerization into account.

## Methods

All molecules were generated via flash pyrolysis in a resistively heated SiC tube from their respective precursor (see Scheme 1). All precursors were heated (**4**: 170 °C, **5**: 80 °C and **6**: 110 °C) to increase the vapour pressure and subsequently seeded in a carrier noble gas (**4**: 1.4 bar Argon; **5**/**6**: 1.5 bar He). A free jet is produced by a solenoid valve and expanded into a differentially pumped vacuum apparatus after pyrolysis at estimated temperatures of 600–800 °C. The jet is skimmed and the adiabatically cooled central part enters the ionization region of a time‐of‐flight mass spectrometer, where it is crossed by the UV and IR radiation. The UV light is generated by a pulsed Nd:YAG (20 Hz) pumped dye laser followed by frequency doubling to produce UV photons of 265 nm for the p‐xylylene and 266 nm for the m‐/o‐xylylene experiments. Laser power was usually in the range of 0.75–1 mJ and the ionization follows a [1+1]‐REMPI scheme. The IR radiation was provided by the free electron laser FELIX (Radboud University, Nijmegen, the Netherlands).^6^ It was scanned over the fingerprint region 550–1750 cm^−1^ in steps of 2 cm^−1^ (**2**/**3**) or 2.5 cm^−1^(**1**) respectively. To maximize the depletion of the ion signal, the UV beam was delayed by 200 ns with respect to the IR radiation. The resulting IR/UV spectra were corrected for laser power, averaged and smoothed using digital filtering. The experimental data were compared to vibrational DFT calculations at the B3LYP/6‐311++G** level of theory,^10^ using the Gaussian program package.^19^ Anharmonic vibrational analysis was performed utilizing the freq=anharmonic option as implemented in the Gaussian09 suite. The resulting stick spectra were convolved with a Gaussian‐shaped function (full width at half maximum=15 cm^−1^). The bandwidth is mostly determined by the rotational envelope, because typical rotational temperatures in our experiments are around 50–100 K. However, some power broadening might be present as well. The excited electronic states and oscillator strengths were computed using EOM‐CCSD^14^ with the aug‐cc‐pVDZ basis set,^15^ also using Gaussian. The structures were optimized using CCSD^20^/cc‐pVDZ^21^ in the frame of the QChem program package^22^.

## Conflict of interest

The authors declare no conflict of interest.
